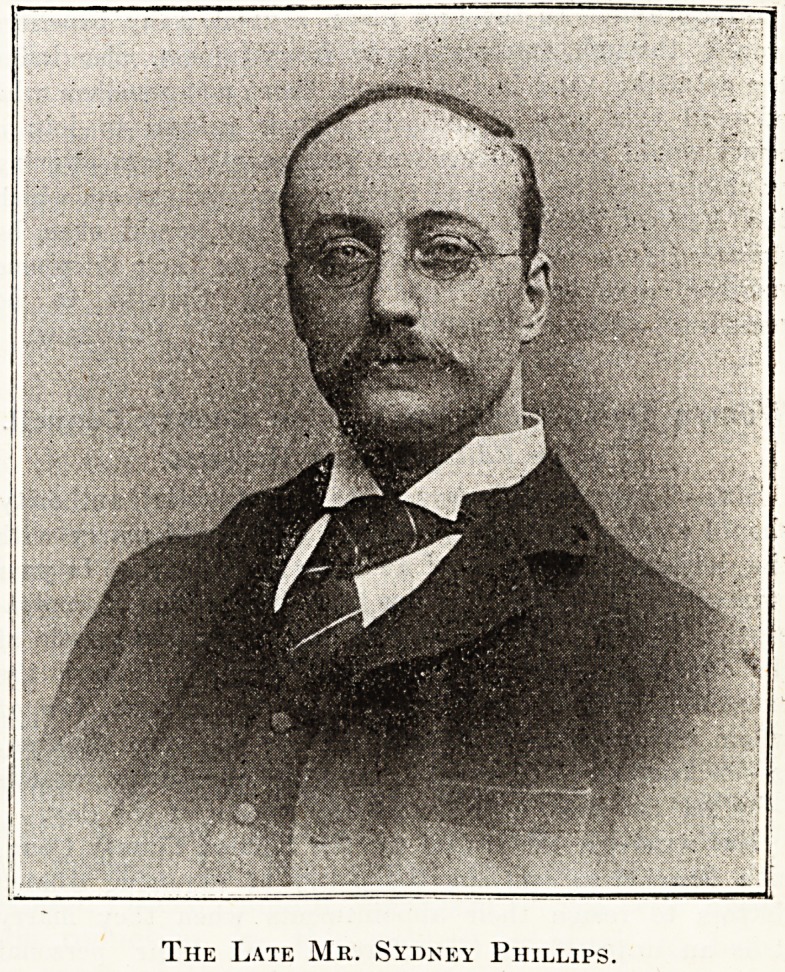# His Work and His Personality

**Published:** 1914-04-25

**Authors:** 


### THE LATE MR. SYDNEY PHILLIPS.

### His Work and his Personality.

SPECIAL MEMOIR BY AN OLD FRIEND.

On Thursday last week, in a quiet corner of Brookwood Cemetery, amongst the pine trees which he loved only too well, the Steward of St. Thomas's Hospital, Mr. Sydney Phillips, was laid to rest. A Memorial Service was held on the Thursday morning in the chapel of the hospital, to which the coffin had been moved on the previous evening from the Steward's house. This service was conducted by the late Vicar of Holy Trinity, Lambeth, the Rev. A. O. Hayes, who was also Hospitaller of the hospital, assisted by the Rev. Mr. Harding. The widow and eldest son, his mother, his brother-in-law, and other relatives, as well as the Treasurer and Miss Wainwright, the late senior surgeon, Mr. G. H. Makins, who had given every care to Mr. Phillips and had operated on him during his illness, Mrs. Makins, Mr. and Mrs. Battle, Dr. Box, Dr. Bevan, Dr. Mennell, Mr. Sargent, Mr. G. Q. Roberts, the secretary of the hospital, Mr. Edmund White, late pharmaceutist of the hospital, and Mrs. White, Mr. Thomas Ryan, secretary of St. Mary's Hospital, Mr. Godfrey Hamilton, secretary of the National Hospital, Queen Square, the Matron, Miss Lloyd-Still, the Lady Almoner, Miss Cummins, and many other officers, sisters, nurses, and servants of the hospital attended.

After the service the body was taken to the Necropolis Station, and thence to Woking Cemetery, where the usual short service at the grave side was impressively carried out by the Rev. Mr. Harding.

### The Story of his Illness.

It is now two years since Mr. Phillips was first obliged to lay up through illness, which he had believed was caused by a very ordinary complaint. Having determined to be operated on he put himself in the hands of the surgeons, but, alas! grave malignant disease was revealed, and a serious operation was necessary. He was practically laid aside for the whole of the year 1912—it was November before Mr. Phillips was able to leave the hospital for a convalescence in Devonshire. He came back a mere shadow of his former self, but still full of hope and anxious for work, in January 1913, and attended to his duties for the earlier part of that year. Further trouble developed and after a small operation Mr. Phillips went to Torquay, and hoped to be back in December. On the very eve of his return came the sad news that, after apparently making good progress, he had again broken down, and been obliged to go into Newton Abbot Hospital, where he was successfully treated under the charge of Dr. Hayden, and the pain relieved by the use of radium emanations, but all hope of recovery had to be given up by his friends, though the patient himself never realised this fact, and when he came back to his home in the hospital in March last it was felt that he would never be fit for more work. He was confined to his bed the whole time, very carefully tended by his devoted wife, and it was not until Thursday, April 9, that Mr. Phillips fully realised that there was no hope for recovery. He died on the 11th inst.

### St. Thomas's and his Early Years.

By his death St. Thomas's has suffered a severe loss, for Mr. Phillips was a good and faithful servant, and an officer fully devoted to the best interests of the hospital. He was elected steward of St. Thomas's in succession to Mr. F. Walker, who had been steward of the hospital for fifty years, no light task for so comparatively young a man. He had had a good training at St. Mary's Hospital under Mr. Thomas Ryan, of whom he always spoke in the warmest terms of affection.

Born in 1868, Mr. Phillips was the son of an old sister of the London Hospital, who was afterwards at Queen Charlotte's Lying-in Hospital, and on leaving the Haberdashers' Company School he first went into the Civil Service, and held a junior clerkship at Greenwich Observatory. From there he undertook the secretaryship of the Hospitals Association, from which post he moved, in 1890, to St. Mary's Hospital, where he acted for six years as assistant to Mr. Ryan. Few, if any, hospital officers have ever shown greater diligence and mastery of the details of their work than Mr. Phillips, for all that he did was marked by accuracy and a knowledge on which the governors of the hospital could place full reliance. During the eighteen years of his work at St. Thomas's great progress was made there. When he first went to the hospital three of the wards were closed, and two were being used only for the reception of paying patients. Now we see the whole hospital in use for ordinary patients and two extra wards built for St. Thomas's Home for Paying Patients. The total number of patients admitted during the year has risen from 6,381, with a daily average of 396, to a total of 9,805 patients treated in 1913, with a daily average resident of 548, while the expenditure in the maintenance of the hospital has grown from £45,960, when Mr. Phillips entered its service, to £76,174 last year.

### His Masonry and Characteristics.

Mr. Phillips joined fully in the social life and gatherings of hospital officers. He had served ably the office of hon. secretary of the Hospital Officers' Club, and was a vice-president of the Incorporated Association of Hospital Officers. At St. Thomas's Hospital he was much respected. He was a Freemason and for many years filled the office of treasurer of the Cheselden Lodge. Mr. Phillips lost no opportunity of turning every hour to some good purpose. In spite of the fact that he had at a very early age to devote himself to work necessary to gain his own support, he yet found time to pass the London Matriculation and to take a good B.A. degree. He ate his dinners and was called to the Bar, and without a doubt the legal knowledge that he acquired was of the greatest value to his hospital. None has better tender forms, and to this branch of his work he devoted great care and ability, the results of which are clearly indicated in the economical administration of the provisions department, which was specially under his care. He was in charge of all the lay arrangements connected with the St. Thomas's Home for Paying Patients. His courtesy in interviewing friends and the tact with which he dealt with difficult situations, which must necessarily arise in a business of this kind, was remarkable and contributed much to the success to which this Home has attained. As secretary of the Samaritan Fund of the hospital he did much for the relief of poor patients on their discharge from the wards, and through his good services many were set up again who, without the convalescent treatment and the aid which this Fund was able to give, might have found themselves surely on the rocks on their discharge from the hospital.

He leaves a widow and two boys, as well as a widowed mother, to mourn his loss. He lived but too short a time, alas! for him to do much to secure the education of these

The Late Mr. Sydney Phillips.

boys in the way in which he had set his heart, and it is hoped that something will be done to give them a chance of making as honourable a career as their father made for himself. As a citizen Mr. Phillips took great interest in the Education Committee of Lambeth, where his services were much appreciated, and, a staunch Churchman, he was a man of remarkable integrity and strict character.

## Figures and Tables

**Figure f1:**